# Has Clinical Translation of Scientific Research been a Failure? Pro

**DOI:** 10.1192/j.eurpsy.2022.44

**Published:** 2022-09-01

**Authors:** E. Binder

**Affiliations:** Max-Planck Institute of Psychiatry, Translational Research In Psychiatry, Munich, Germany

## Abstract

While current psychiatric treatments, especially pharmacological ones, are mainly based on initially serendipous findings, recent developments in many research areas promise unprecendented advances in translation to clinical application. The advent of large scale multiomics with single cell resolution, patient-derived induced pluripotent stem cells that can be differentiated to brain cells and even form cerebral organoids as well as the possilibility to generate humanized animal models using CRISPR-technologies have been exciting developments on the molecular and cellular side. These advances are paralleled by new technologies to increase resolution in brain imaging and electrophysiological measures as well as brain stimulation techniques. In addition, we have now access to multilevel data from very large cohorts, increasingly with longitudinal measures and using data collection with digital devices and wearables. Artificial intelligence methods open new possibilities to analyze these large scale, multilevel datasets in unbiased ways. This side of the debate will highlight the translational potential of these advances in research possiblities, giving examples from the areas of novel pharmacological treatments and personalized medicine among others. Furthermore, it will highlight how a better understanding of risk factors, genetic, social and environmental, can lead to improvements in prevention programs.

**Disclosure:**

No significant relationships.

